# Prevention of HIV and Other Sexually Transmissible Infections in Expatriates and Traveler Networks: Qualitative Study of Peer Interaction in an Online Forum

**DOI:** 10.2196/10787

**Published:** 2018-09-05

**Authors:** Gemma Crawford, Bruce Maycock, Rochelle Tobin, Graham Brown, Roanna Lobo

**Affiliations:** ^1^ Collaboration for Evidence, Research and Impact in Public Health School of Public Health Curtin University Bentley Australia; ^2^ Australian Research Centre in Sex Health and Society La Trobe University Bundoora Australia

**Keywords:** HIV, STIs, men, public health, health promotion, online social networks, social support, travel, human migration, emigration and immigration, sexually transmitted diseases, social networking

## Abstract

**Background:**

In high-income countries such as Australia, an increasing proportion of HIV cases have been acquired overseas, including among expatriates and travelers. Australia’s national strategies have highlighted the need for public health interventions for priority populations. One approach is to expand efforts to places or spaces where expatriate communities reside. Online settings such as forums used by expatriates and travelers have potential for preventing sexually transmissible infections with those hard to reach through more traditional interventions.

**Objective:**

Our objectives were to (1) identify and describe domains of social interaction and engagement in 1 online forum used by Australian expatriates and travelers living or working in Thailand; and (2) make recommendations to health-promoting organizations and policy makers regarding the role of these forums in public health interventions with mobile populations who may be at risk of acquiring HIV or other sexually transmissible infections.

**Methods:**

We identified forums and users in 2 stages. We identified 13 online forums and analyzed them for inclusion criteria. We searched 1 forum that met the required criteria for users who met inclusion criteria (n=5). Discussion threads, rather than individual posts, were units of analysis. For each user, we collected as transcripts the first 100 posts and 10 most recent posts, including the thread in which they were posted. We analyzed and thematically coded each post (n=550). Transcripts and analyses were reviewed and refined by multiple members of the research team to improve rigor. Themes were not totally emergent but explored against symbolic interactionism concepts of presentation of self, meaning, and socialization.

**Results:**

Key domains were as follows: the forum (characteristics of the space and reasons for use), gaining access (forum hierarchy and rules), identity (presentation of self and role of language), advice, support, and information (sources of information, support provided, influencers, topics of discussion, and receptiveness to advice), and risk (expectations and perceptions). The forum exhibited evidence of unique language, rules and norms, and processes for managing conflict and key influencers. The forum was a substantial source of health information and advice provided to users via confirmation, reassurance, or affirmation of beliefs and experiences. Risk perception and expectations varied. Risk taking, including around sex, appeared to be a key expectation of travel or the experience of being an expatriate or traveler.

**Conclusions:**

Australian expatriate and long-term traveler participation in the online forum formed, influenced, and reinforced knowledge, attitudes, interaction, and identity. Such forums can be used by policy makers and health-promoting organizations to provide supplementary sources of support and information to hard-to-reach mobile populations who may be at risk of acquiring HIV or other sexually transmissible infections. This will complement existing engagement with health professionals and other public health interventions.

## Introduction

### Overview

In high-income countries, including Australia, population mobility has led to changes in transmission patterns of HIV and other sexually transmissible infections (STIs). An increasing proportion of diagnosed cases have been acquired overseas, including among expatriates and travelers [1-5]. Developing interventions to respond to these increases is challenging in part due to geographic barriers to those affected. Online settings can overcome such difficulties. We explored social interaction and engagement in 1 online forum used by Australian expatriates and travelers living or working in Thailand who may be at risk of acquiring HIV or other STIs. We sought to determine the possible uses of such forums for public health intervention, providing new insights for health-promoting organizations and policy makers working in sexual health with mobile populations.

### Background

Migration and mobility are inevitably connected with changing environments. Expatriates and travelers may experience a high degree of liminality (described by van Gennep and Turner as transition, a sense of being “being betwixt and between”, as reviewed by Thomassen [6]) within and between environments, raising issues of identity and belonging. Research by Brown and colleagues [4] with Australian male expatriates and long-term travelers residing in Southeast Asia suggested that identity and behavior were strongly influenced by local social networks. Support and guidance on how to adapt to the social and cultural norms were gained from peers.

Such findings have implications for the way that countries develop their response for HIV and STI prevention, treatment, and care. Australian frameworks have highlighted the need for public health strategies to target priority populations, including men who travel overseas frequently for work or leisure [7]. One way this may be achieved is to expand intervention efforts into places or spaces where expatriate communities reside [2-4,8], including online settings such as forums used by expatriates and travelers.

Online spaces provide a medium for education and prevention, an approach used effectively with marginalized or vulnerable groups in areas such as youth mental health [9] and public health interventions with gay and other men who have sex with men [10]. Such settings can enhance social capital and community connection and decrease social isolation. This is particularly the case for those who may be difficult to engage or access through more traditional communication methods, or for those who may not identify with general health promotion messages [3,10-13]. These methods may also reduce socioeconomic or geographic barriers caused by stigmatization and afford some level of anonymity to individuals seeking support or information online [10,14-17].

For mobile populations such as expatriates and long-term travelers, connection online may reduce perceived and actual distance between country of origin and destination. Such spaces may reduce some of the liminality experienced or create a “home away from home” [18]. Online communities facilitate peer influence as platforms for individuals to exchange social, emotional, and informational support, share experiences, and seek advice [19,20]. These functions may prove useful in regard to health advice, resettlement, and language, as well as contributing to a sense of belonging [21,22] or a deepening connection to the destination country and others within the peer and social network [18,23].

### The Study

There is a lack of literature describing the online information-seeking behaviors of expatriates and other long-term travelers and how advice from their interactions with one another online may influence risk and protective behaviors. This study built on our understanding of Australian expatriate and long-term traveler risk behavior, culture, and experiences [3,4] and the lessons learned from previous successful use of peer influence models with communities and populations at risk for acquiring HIV and other STIs, particularly in Australia [24].

This paper describes an in-depth analysis of social interaction in 1 online forum used by Australian expatriates and travelers in Southeast Asia. We identify the way in which the forum functions as an online community, describing engagement between users; user identity and how the forum mediates this; types of advice and information shared and acceptance of that advice; and perceptions of risk. We make recommendations for policy makers and health-promoting organizations to use these findings to develop, improve, and expand the reach of public health interventions to reduce the transmission and impact of HIV and other STIs with mobile populations who are hard to access.

## Methods

### Overview

This research was part of a larger qualitative study to determine whether the social networks of Australian male expatriates and travelers in Southeast Asia can support strategies to reduce or prevent the transmission of HIV and other STIs [1]. The focus of this research was to develop greater understanding of Australian expatriate and traveler culture, behavior, and socialization and the potential for members of the target group to act as social influencers around knowledge, attitudes, and behavior. We used conversations from online forums as (1) a source of data and (2) an audit of spaces that expatriates and travelers frequent to assess the online environment for its potentiality for intervention.

### Conceptual Framework and Methodology

Symbolic interactionism provided the conceptual framework underpinning this study, as it has useful application to public health [25] and to sexuality and HIV specifically [26]. The symbolic interactionism perspective supports the idea that social interaction is used to construct reality and that individuals interpret and respond to objects and others’ actions based on meaning that is created by interaction [27]. Analyzing forum discourses in this way provided insight into how individual attitudes and behaviors were influenced through social interaction. Charon [28] suggested that symbolic interactionism allows for exploration of the development of self and self-identity and how this is influenced through social interactions. We used this point of view when exploring the transition from novice forum user (newbie) to experienced forum user (expert) and to understand how individuals may come to self-identify as an expatriate or long-term traveler.

### Research Team

The research and authorship team was composed of 5 members experienced in public health research. Of these, 2 were students at the time of writing. Several members of the research team had experience working in community bloodborne virus organizations, while others had experience working with marginalized or vulnerable groups through qualitative and participatory action research. All members of the mixed-sex team had spent time in Thailand, with 3 of the members collaborating on previous research in Phuket at the commencement of the research project. Members of the team were also experienced in conducting research in the use of online strategies for public health [14,29-31].

### Selection Criteria and Forum Search Strategy

**Figure 1:**
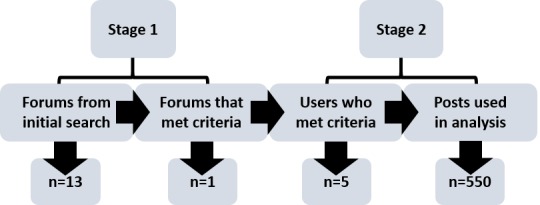
Process of identifying users.

We undertook a comprehensive internet search over a 2-month period to identify forum users for inclusion in this study (Figure 1), described below.

In the first stage, we identified online forums frequented by the target group (Australian male expatriate or long-term traveler to Southeast Asia) through an internet search. At the time of writing, there were at least 10 online forums with thousands of members that Australian male expatriates and long-term travelers were using.

We determined the internet search terms by reviewing commonly used terminology on a variety of forums and a thesaurus search, guided by a review of the literature. Members of the research team provided consensus on search strategy terminology. We entered search terms into Google (Google LLC, Mountain View, CA, USA; Textbox 1) and identified a total of 13 forums.

Search outcomes were checked by 2 members of the research team to ensure consistency. We selected potential forums according to a list of predetermined criteria (Textbox 2). These included whether the forum met the criteria for an online community as operationalized by Herring’s 6 dimensions: (1) active participation, (2) shared culture and norms, (3) roles, rituals and hierarchies, (4) a distinct identity, (5) solidarity, and (6) support and conflict resolution [32].

Of the 13 forums, only 1 met the selection criteria and was included in this study. We excluded other forums because (1) we were unable to access a sufficient number of posts, (2) the nationality or sex of users was unclear, or (3) sufficient data were not publicly available.

In the second stage, we searched the identified forum for users who self-identified as Australians, who had resided in or were currently residing in Thailand, and who had created over 100 posts (determined to be a sufficient number to enable the analysis of the socialization processes along the trajectory of forum user newbie to expert [33]). Using these criteria, we identified 5 users for inclusion, and we considered their posts and interaction with other members for data collection.

**Google search terms.**

(Forum OR blog OR chat room) AND (Expat* OR foreigner OR ‘long term traveller*’ OR ‘permanent tourist’ OR ‘permanent resident’) AND (Thai* OR ‘South East Asia’ OR South Asia OR Southasia) AND (Australia* OR Aussie OR Oz)

**Forum inclusion criteria.**

Posts are accessible to the public.

Users include Australian males who have resided or are residing in Thailand.

Allows identification of the nationality and sex of users.

Allows individual user’s post history to be tracked.

Conforms to Herring’s definition of an online community [32].

### Ethical Considerations

Ethical challenges related to conducting research within online communities include how, whether, and from whom informed consent is gained; whether anonymity can and should be protected; and sensitivities relating to communities that may discover they are being “researched” [34]. We reduced the risk of altering online discussion based on our presence by not making ourselves known on the forum and not becoming members. This was consistent with other studies where researchers have taken on a remote or objective role and allowed conversations to continue unhampered by the presence of an outsider [33]. Curtin University, Perth, Australia, provided ethical approval for this study. In line with the requirements of the institutional ethical approval and to protect the integrity of the forum and the anonymity of users and their contributions, we have not identified the forum and have deidentified individual users.

### Data Collection

Because a forum user’s post rarely occurs in isolation, meaning would be lost if the post is not considered within the context of the surrounding discourse and interaction with other users. Consequently, we collected both posts and threads. We took the definitions for threads and posts from Arsal and colleagues: (1) threads are “hierarchically organized postings.” and (2) posts describe, for example, “a message written in the online community forum” [22]. For each of the 5 included forum users, we collected their first 100 and 10 most recent posts, along with the thread in which they were posted. This involved using the forum member list to search for the profile of each user and then accessing their posts and threads. We then captured the posts and threads as transcripts of activity.

### Analysis

Discussion threads on the forum, rather than individual posts, were the units of analysis. We imported transcripts into NVivo 10 software (QSR International Pty Ltd). Each post was analyzed and coded thematically by a member of the research team, and a sample of transcripts was validated by a second researcher. This process continued until each of the 5 users’ posts (n=550) had been coded thematically. Then, 3 research team members reviewed transcripts, compared analyses [3], and developed and refined themes. The remaining team members reviewed samples to improve rigor. Themes were not totally emergent but explored against symbolic interactionism concepts of presentation of self, meaning, and socialization [28], with a focus on interaction within the forum.

## Results

The following were the key domains produced from the analysis: *the forum* (characteristics of the space and reasons for use), *gaining access* (forum hierarchy and rules), *identity* (presentation of the self and the role of language), *advice, support, and information* (sources of information, support provided, influencers, topics of discussion, and receptiveness to advice), and *risk* (expectations and perceptions).

### The Forum

The forum was well used by Australian male expatriates and long-term travelers. Relatively new at the time of writing, it had over 900 members internationally and in excess of 300,000 posts and 7000 threads. Public information included a member list presenting the member handle and avatar, user join date, number of posts, and last visit to the forum. Data on user numbers at any given time (and status as either member or guest) were also available. It was common for the forum to have several hundred active users at any time, with peak use involving several thousand users (comprising members and guest users). Guest access provided limited viewing access to publicly available spaces within the forum. Member access provided users the ability to post on topics, communicate privately with each other, participate in polls, and upload content.

The forum facilitated a space where those visiting or living in Thailand could seek and provide advice, and establish and maintain online and offline social networks. Forum topics included visas, language, navigating cultural differences, health, sex and relationships, where to go for a night out, and how to avoid being “ripped off.” Users demonstrated an interest in building a community of like-minded individuals, which appeared to be an expected and enforced 2-way interaction, as evidenced by a post from a member:

Whilst forums can be supportive of different points of view, in my view they ultimately work because membership is primarily comprised of like-minded individuals that want to pursue similar goals...In (our) case, it is about genuine desire to understand Thai culture, and give it due respect.

This sense of reciprocity was reinforced by the forum moderator as critical to new users:

...part of the forum is the giving and receiving of information, it is maybe how we learn or get to know someone.

Users displayed protectiveness toward and ownership over the forum:

We are passionate about the Forum so some people will react to some things said as we are all trying to protect what we have here...if you are just Trolling, this is not the place for it.

Staying connected to each other, and to Thailand, while in different parts of the world appeared to be the primary reason for using the forum. For example, users who now lived in Australia used the forum to “cure the LOS [Land of Smiles, slang for Thailand] blues.” Social ties were developed and enhanced through forum use. As a user commented, “It is great how close everyone has become over a Forum. I cannot wait to continue meeting everyone and sharing my story as it goes on.” Seeking connection was exemplified in the posts of 1 user, who suggested the forum provided a way to “kill the loneliness” and meet new people when in Thailand. Quick to extend invitations to new and old users to meet in person, he used extensive knowledge of Thailand, including language and music, to provide advice and establish a connection:

You sound interesting. You also sound like you would welcome some tips and tricks over a few drinks...I lived in Bkk [Bangkok] for 11 years, speak the lingo but now spend just two months a year there.

Several users posted prolifically but tended to avoid sharing personal information and instead offered advice on a variety of topics, such as visas, motorbikes, and relationships. The owner of a pub, who also appeared to know other users offline, used the forum to promote his business, without appearing as though it was advertising. He offered himself as a source of local knowledge, noting “I’ll help any visitors as much as possible.”

### Gaining Access

A hierarchy was evident on the forum, with 3 levels of access ranging from publicly available threads and posts to higher-level, invitation-only spaces, not openly accessible. This hierarchy and structure provided users with the opportunity to become more involved and more deeply connected with the forum and peers.

Level 1 was public; members and nonmembers were free to read posts and contribute posts once they had created an account. It contained general information on life in Thailand, such as relationships, visas, and travel advice. Posts of a sexual nature were not permitted. As the forum moderator described, “P4P is not a focus here so keep it general, if you want prices here is not the place.” P4P is a term used to describe pay for pleasure, sexual services provided for money. Private threads in the upper levels containing useful and appropriate advice for the public were generally moved by moderators to level 1.

The second level was accessible once a user had made 30 posts. Users shared more personal information, including photos and details of relationships and sexual behavior. It was described as a place where you could “get down to the nitty gritty.” A new user deemed “one of us” would be quickly welcomed and encouraged to post more so that they could access this level. This occurred when a user posted for the first time and was told:

A few more posts and a whole new world will open up on here.

An experienced guy like you...you’re [*sic*] input will be welcomed...

You are 3 posts off getting to a totally new world.

The third level was by invitation only. One of the users, realizing he did not have access after posting on level 1 over 50 times, asked how to gain access and was told to send a private message to the moderator as “It’s a secret handshake not a post count.” He then gained access (“I’ve been admitted to the secret society. Now I’m off to practice that handshake ;)”) and was told by another forum user to “enjoy and contribute...some smut 555” (5 in Thai is pronounced *ha*).

An administrator oversaw forum operation along with 2 moderators. Rules guided forum behavior, which were rarely but explicitly spelled out for users:

...no personal attacks will be tolerated!! Any personal abuse will be deleted. Repeat offenders will be given a yellow card [sporting reference relating to the use of a yellow card to caution a player about their behavior].

Interaction demonstrated clear self- and peer moderating. Users reinforced expected behavior regarding contributing and valuing opinions and respecting Thai culture. Users quickly excluded new users who did not meet rules:

If you are here to play games...well we are not game players and we will just go quiet. Welcome to the Thai way. We just go quiet.

Those familiar with the rules quickly resolved miscommunication or disagreements. An example of this was when a user posted a link to discuss with other members but did not contribute his own opinion. Another user challenged him, “May I ask what your problem is? You post a vid, people are responding to it, without any contribution of your side”. The first user responded, “You are right it is a discussion and I have not offered an opinion.” He attempted to prevent further miscommunication, “I do enjoy your posts, which show a keen understanding of the human condition. I think...wow there are some smart dudes on this forum.”

### Identity

Users decided on their presentation of self, creating online avatars and identities, and providing data establishing their credibility and belonging. For example, one user described himself as a “no-one in Australia and a VIP in Thailand.”

Each of the 5 included users identified as Australian, were proud of their culture, and identified as “Aussies in Thailand.” Posting in the forums reinforced this sense of Australian identity in relation to law, food, sport, or society, for example, a posting about Australian-style bars: “Chiang Mai’s only genuine big Aussie pub...Great old fashioned Aussie style hamburgers and more...”

For some users who traveled back and forth between Australia and Thailand, there was a clear delineation between their identity at home and abroad:

Thailand is sort of like my “what happens in Thailand stays in Thailand.” Two weeks of partying real hard then back to the “real world” as you call it.

Posters used a combination of Thai and Australian-English slang that appeared unique to the English-speaking expatriate community and, most particularly, Australians. Users explained the meaning of phrases they used when asked, and a specific thread covered basic abbreviations, slang, and the use of ideograms or emojis (this was one source of information for us regarding terminology with which we were unfamiliar). For example, while ATM was used for “at the moment” it was also used to describe “a man who dispenses cash to a TG [Thai girlfriend] or BG [bar girl].” The use of this specific language appeared important in establishing commonality and determining how quickly new members were accepted:

Heh heh you sound sufficiently deviant 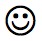.

In his first post, a user presented himself as being “one of you,” using language demonstrating he had spent extended periods of time in Thailand and knew the language well, ensuring he would have a role to play on the forum as a source of advice. He used language that would be familiar to other users, such as *falang* (Thai for a foreigner of Western descent, also often written as *farang*).

A similar approach was taken by another user introducing himself as an “Aussie pervert who loves motorbikes and football.” He was immediately accepted and received welcoming comments, such as “well you tick all the boxes 555...WTTB [welcome to the board].” It is important to note that the use of the word pervert here is culturally specific and used to describe broad sexual interests in a humorous way, but it does not necessarily relate to a technical or formal definition of pervert, particularly where it might relate to illegal sexual activity.

### Advice, Support, and Information

Users gave and received a variety of information and advice and provided different levels of support to one another. Posts under the topic heading Trip Reports shared the ins and outs of recent travel, including sexual encounters. Users learned and shared through stories of caution, romance and relationships, sex, mentoring, health risks, and culture, which created commonalities and built rapport.

Discussions were often based on what was reported in the news, with an avatar created specifically to post about news. Users were quick to incorporate statistics, anecdotes, or news from a variety of sources into discussions with varying levels of accuracy and evidence. Demonstrating the power of a cautionary tale in mediating behavior, a user posted in response to a story about a fatal road crash:

I love cruisin’ around the provinces during my Thai holidays, but have always been aware that the risks are so much higher than in Oz. Hearing of this tragedy only makes me so much more aware. I always intend to travel on during daylight hours but sometimes drive into the night to get to a desired destination. I think I will now take more care in planning my times of travel and be ever mindful to drive defensively.

Some users acted as influencers, encouraging participation from others (the first 50 users to post were given the title of Founding Member):

We, the founding members, can only impart so much knowledge, experiences and advice. The forum needs the input of others, like yourself to cover all the bases needed.

Key individuals held roles as “sages” (eg, “I was at [X’s] ‘Table of Wisdom’ (555) y/day afternoon...,” referring to an individual and their bar and the way in which they “held court” in that space). In this way, they told stories about the support that they had provided for newbies. This established or reinforced their role as a credible expert:

Well he was really intrigued, but I could see he was a bit out of his element, so I asked if he wanted to meet up at our hotel that night, and we’d introduce him to [the area]. He was all for it...we had a blast both that night and last night...The funny part was, when we saw him that first night he said “I want you to know, you have successfully mentored me!” “What do you mean?” I said, “Well” he says, “I got a massage today, and the girl giving me the massage was really nice, so I asked her out to dinner, and we’re going to see the elephant show as well, and she’ll be at the boxing with me tomorrow!

In relation to romantic or sexual relationships, advice sought and information shared was often explicit and detailed. A new user described his experience:

In hindsight...after my sickness I seemed to totally lose all sex drive. It wasn’t at all like me...From then on I became more a peaceful observer rather than an active hunter. The more the “sex sell” was offered the further I felt pushed away. I became too aware of the business side of things, the desperation and felt sorry for some of the girls’ situations. It was like being at a disco when all the lights are turned on and the music stopped, the vibe dies and certain realities become more apparent...Probably just need to spend more time on the prowl and have a bit more determination? Having a GFE [girlfriend experience] would have been nice, but I didn’t have much expectation. As a result I only packed 3 boxes of condoms of which none were used. 555

In response, another user provided advice and empathy, establishing commonality and solidarity:

Mongering [loosely defined in this context as seeking sex] isn’t for everyone. It seems like you enjoyed your holiday, but if you ever come back, see about finding a wingman. I think that will make it a lot easier and more enjoyable for you to go out.

Users appeared generally receptive to and accepting of advice and information provided by others on the forum, often explicitly seeking it. For example, a new user posted:

Thanks for the replies gents, and for not ripping me a new one for poor searching of the forum! Managed to get one night with a couple of other like-minded individuals...am very familiar with the P4P scene and frankly love it. What I really want to find out...any BJ [slang for blow job, oral sex] bars or decent massage parlors for a bit of light relief...?

In response another user posted:

I’ve never found a BJ bar, but there are dozens of massage shops all over...you can casually wander up and down til you find a spot you like the look of. Any more info than that and I’d be spoiling the adventure.

### Risk

Experienced users reinforced a liminal space of adventure and temptation. The level of or willingness for taking risks seemed to be based on active decision making, previous experience, location, advice from others, and the role of luck or fate. For some, risk was considered to be part of the reason for travel (or being an expatriate or long-term traveler), while for others risk was an expected byproduct of the travel. Users discussed and described a range of issues, including untrustworthy airlines, motorbike use, road use, scams, travel insurance, or STIs: “I wonder if travel insurance would cover you if you got HIV or some other STD [sexually transmitted disease] overseas 555.”

Expectations were presented around the exotic and erotic, suggesting generally permissive attitudes toward time away. Sex and alcohol and other drug use was normalized as part of the expatriate or traveler experience:

Other than the great food, weather and beaches why not top it all off with something you can’t do at home? Walk straight into a bar, pick up a chick usually much younger than yourself and go home and have fun all night long? Eat sleep boom boom REPEAT!!!

Self-control seemed to underpin risk taking or risk management for some (eg, “I had a sober week out of the three last trip...stuck to soda waters but they were still trying to give me shooters as well”) with users frequently describing that “temptation is not far away in Thailand...” requiring moderation and discipline:

One of the biggest hurdles living in LOS [Land of Smiles]is all the temptation whether it be the girls, the food, the partying it’s all got to be done in moderation or health and weight problems creep up on many expats I’ve known here.

A sense of frustration toward those perceived as “not following the rules” was exhibited by others. These appeared to be generally accepted and known, and legitimized the identity of expatriates or long-term travelers, differentiating them from other vacationers. Personal responsibility, luck, and karma featured in many descriptions of risk taking:

What is it about being on holidays that warps people’s minds?? They go off and do things they wouldn’t normally do at home. Hire a bike or scooter and take on roads they know nothing off and no knowledge of local driving in one of the most lawless drivers in the world...but hey...I’m on holiday so let’s do it!!! Jump off cliffs, hire a jet ski and ride like idiots, hire a prostitute and go bareback...but it’s holiday time...FFS [for fuck’s sake]!! Then when they come undone it’s everyone else’s fault.

Condoms were mentioned with regularity, with discussions relating to frequency of use, use with different partners, and the efficacy of different condom types:

Except for one occasion I have never gone bareback and use condoms always. Never had an STI either, maybe more good luck than anything else.

There appeared to be a range of knowledge and understanding or concern regarding the difference between pregnancy prevention and STI prevention, and interventions to address these issues: “Speaking of condoms...I ALWAYS used them when I had sex with a woman who was not taking the pill. Never had a failure...”

## Discussion

### Principal Results

Interactions illustrated complex processes of socialization, acculturation, and identity formation and presentation among Australian expatriates and travelers. Key themes emerged regarding advice and support, perceptions, and expectations around risk taking, which have particular resonance relating to prevention of HIV and other STIs. A large number of users were active at any one time, as well as a range of other viewers, who may have included observers, trialists, or those seeking information rather than the reciprocity inherent in greater participation [35].

The forum functioned as an online community providing a space to share common interests and confirmation, reassurance, or affirmation of beliefs and experiences. There was evidence of unique language, norms, and processes for managing conflict [32]. This self- and peer-moderating behavior demonstrated a peer network with clear rules that created and reinforced culture. Users exhibited intense loyalty toward the forum, which, consistent with findings by Hiller and Franz [18], suggests development of a nascent identity rooted in distinctive language, rituals, folkways, and collective network consciousness.

Key influencers emerged, including those with formal roles, such as moderators and longer-serving, high-posting members, who may, as Kavanaugh and colleagues [36] have suggested, be considered bridges in the community, capable of expediting information distribution. We noted layers of complexity, with some users interacting not only in general forums, but also in social spaces outside the view of the public, including offline, other forums, and members-only sections. These interactions appeared to enhance social connectedness, building and augmenting online and offline relationships [37].

The forum provided significant social support, information, and advice about certain health issues, including HIV and other STIs. This was both directive (practical advice) and nondirective (sharing personal experiences) [38] and in particular focused on informational and emotional support. While many users may have initially joined the forum seeking information, participation continued due to the relationships formed with other members. Risk perception and expectation among users varied. For example, it was clear that, despite sharing stories of risk behaviors, some users did not consider HIV and STIs to be personal risks. Further, much of the information provided about these issues was based on anecdote and word-of-mouth. It appeared that, for many, risk taking, including seeking sexual services or trying something new sexually, was a key expectation of travel or the experience of being an expatriate or traveler.

### Comparison With Prior Work

Cultural norms and rules influence the operation of communities and networks [39]. Previous research examining sexual and social networks of men who have sex with men and HIV risk suggested that common norms regarding risk characteristics and behaviors are created [40]. Communities, such as this forum existing predominantly online, gradually develop norms as members interact and debate and agree on what is acceptable [41]. We found sophisticated governance regarding acceptable behaviors and a preestablished network with sustainability and structure. These elements are important to the strength and stability of a peer network, and are important considerations for intervention using a social network or peer approaches such as those used with people who inject drugs [42,43] or men who have sex with men [40].

Members mediated the behavior of new users, and the moderator enforced or reinforced group norms. This generally appears to be the case in online forums, where rules and norms reduce unwanted behavior [44,45]. In this case, it is unclear whether these rules and norms deterred people from joining or inhibited contributions to the community; however, based on the number of users and overall posts, the impact was likely minimal and may have been a way to filter out those less likely to participate “appropriately.” Additionally, the forum encouraged registration to access greater levels of privilege and a perceived period of probation in which behavior of new members was observed and supported (or not) [46]. This is consistent with other literature suggesting that the use of reputational and trust metrics can support the management of online communities and prevent or reduce abuse [32].

Studies suggest that key features mediating the success of online communities include trust, honesty, and reciprocity [47-50]. We found that reciprocity was an expectation in this forum, for example, where users read messages in a thread but didn’t post and were subsequently criticized as not contributing in the spirit of the forum. A study on influences of consumer behavior in online travel communities concluded that travelers were more likely to follow advice if the online community was trusted and if information provided was perceived to be useful [51]. Kavanaugh [52] has suggested that online networks can build two kinds of trust within groups, defined by Putnam [53] as thin trust (not as personal and established through social relationships that are indirect) and thick trust (triggered by intensive contact among members). The results of this study found evidence of both thick and thin trust with frequent, high-intensity participation by some members, including moderators and founding members, as well as infrequent participation by those seeking information or participating in the lower levels of the forum.

Communities and groups all contain individuals who influence others and who are often explicitly named and rewarded [45,54,55]. On this forum, they were named founding members, gaining access to more private levels after posting frequently, or were given a title of moderator, defined by the forum as “users who are particularly helpful and knowledgeable in the subject of the forum they are moderating.” In this way, they could be seen as influencers or opinion leaders. It has been suggested that engagement, positivity, and effective support may be gauges of influence [54,55]. The use of influencers in interventions is an effective vehicle to communicate information in a manner deemed culturally appropriate to peers, who will in turn more readily receive such information or support [56]. This is a model described in the literature in relation to diffusion of innovations relating to HIV prevention or risk within a network [57]. We found that this community demonstrated many similar characteristics. This is consistent with positive outcomes from historical network-level studies indicating the effectiveness of opinion leaders and peers [58,59] in reducing sexual risk taking and in studies exploring the positive impact of peer support for men living with HIV [60,61].

Community connection can play a significant role in reducing stressors connected with migration, providing a social support system, which can reduce psychological distress or culture shock [62]. Our study found a range of advice, information, and support provided and sought. Interaction influenced knowledge and behavior related to health (including risk taking and health protection) and relationships, as well as the migration experience. Our results resonate with those from other research [38,63-65], including in the context of Web- and peer-based interventions examining mental and sexual health promotion targeting men who have sex with men and same sex–attracted young people [29].

Cutrona and Suhr [66] have proposed a system of social support categorization comprising emotional, informational, social network, esteem, and tangible support. Consistent with this categorization, we found evidence of all forms of support categories, particularly informational and emotional support. Previous studies suggested that members in online communities who receive emotional support will remain members longer than those receiving only informational support [67] and, further, that disclosure is more likely to elicit emotional support than question asking. Our study, consistent with others, found that informational support accounted for a large proportion of interaction [65,68] posited to be because users participating in specific topic forums have similar interests or problems [69]. Online support can be empowering for individuals, and sharing stories can affect health behaviors, including self-care and help seeking [63,70,71]. However, as we found to some extent in this study, peer support may also reinforce perceived unhealthy behaviors or norms or may influence others to make more risky decisions [72].

Forums can be a source of health information as well as a conduit for such information [63]. We found that users critically considered the information presented, engaging with and using advice and support, which most resonated with personal experiences [73]. Consistent with other studies, the information provided by other men in the forum (peers) appeared to be well considered, often more highly valued than advice from health professionals or expert news sources [70,73]. While personal narratives may not always be reliable and in fact have iatrogenic effects [70,74], studies suggested that most information presented in forums is actually of relatively good quality [63]. This reinforces that forums are effective platforms for dissemination of health information and that peer information based on personal experience is considered generally trustworthy [73].

“Communification” has been described as connecting an individual to a community involving a process of meaning making through communication of symbols that can arouse strong attachments [75,76]. This forum exhibited features described by Baron and others as particular to online communication with stylistic and technical peculiarities contributing to the creation of a specific and unique language, credited as important in building solidarities [77,78]. This presented through the use of slang, humor, and ideograms unique to forum-using expatriates. We found that users maintained a strong sense of Australian identity despite significant time spent in Thailand, with Australia as a symbolic anchor [18]. Members exhibited a keen sense of place and identity—as “Aussies in Thailand”—with related loyalty to place of origin and new contexts. Thus, while the forum served to sustain old ties and contribute to new identities [18,37], it may also have contributed to homogenizing or reifying cultural differences, which could be counterproductive to migration, reducing social mobility or acculturation or reinforcing social norms that may be deemed unhealthy.

Posts highlighted how users presented self. These findings are consistent with observations by Goffman and others who suggested that, when interacting socially, individuals put on a “front,” or create an idealized self, aimed at managing impressions and perceptions [79-81]. Users sought information from others in order to determine how interaction occurred, using that knowledge to portray a version of self that was acceptable to others and that reduced the likelihood of role clashes. As with other research [81], we found that, even when there was connection between online and offline spaces, users spent time creating the identity they sought to present to others by managing the information they shared with other forum users. We found, similar to others [72,82,83], that users took on a range of roles and participative stances, with evidence of protagonists, experts, befrienders, and lurkers [35,46]. Our findings, as in the broader literature [46,54], found that the more charismatic characters helped to draw out others in their participation by providing mentorship and “wisdom.”

### Strengths and Limitations

To our knowledge, this is the first study to investigate Australian male expatriates’ and long-term travelers’ social interactions within an online setting, particularly from a public health perspective. The observational nature of this research was a strength. Analysis of publicly available content allowed us to witness real-world interaction unobtrusively. The influence of our presence was removed, allowing individuals to communicate openly in the online environment [16]. However, by remaining invisible, we were unable to pose direct questions or comments that could elicit posts relating to aspects of the broader study, in particular specific knowledge, attitudes, and risk behaviors of users associated with sexual health and STIs.

While we acknowledge that posts from a single forum cannot provide definitive accounts of all aspects of the lives of expatriates and travelers, it was a large and valuable source of naturalistic data [13]. A range of expatriates and travelers were represented with different profiles and demographics (eg, different ages and relationship status; regularity of posts; experience with travel; and social and business intentions), which, while not intentional in sampling, was a useful outcome.

Pragmatic considerations meant that we collected posts in a limited time frame (around 8 weeks). However, analysis of the first 100 posts [33] and last 10 posts, and consideration of the interaction within threads, allowed for exploration of socialization over time and levels of engagement between several users (15 to 20 or more). We encountered difficulty accessing information “behind the wall” in the higher levels of the forum, relying on publicly available information and information in the lowest level of the forum, along with general accounts information located in the higher forum levels. What we did find in the lower level of the forum, however, was a range of information and interaction that was relevant to the study, particularly as this would be the level most accessible to those most in need of information.

The nature of the research meant that, when the meaning or context of posts was unclear, we were unable to seek further clarification. However, the use of language including slang, emojis, and avatar identities provided some further insight into how users presented themselves to others.

Users chose how much personal information to share in forums and how they would present themselves. Additionally, users who posted and responded to personal stories might be different from those who did not. However, the comparative anonymity online and the high degree of trust and credibility that was evident suggest that users shared a significant amount of honest information about themselves, particularly where users were connecting with one another both online and offline.

### Implications for Health Policy and Practice

The study provides an important contribution for policy makers and health-promoting organizations in sexual health looking at opportunities, or unsure how, to adapt community and network engagement strategies to this emerging area. Our findings support the limited research insight about these networks and communities and the way they interact or build community. This knowledge is key to identifying and developing or adapting strategies. As an example, mobile populations are named in the Australian national strategy [7] as a priority group, but little clarity is provided for organizations or policy makers in how or where to respond, nor has there been until recently a solid synthesis of knowledge in this area.

We suggest several considerations from this research for the development of policy and interventions to access this hard-to-reach group that is vulnerable to HIV and other STI transmission. These relate specifically to intervention design, evaluation, and future research.

#### Intervention Strategies

Participants in the forum provided and received social support, and influenced one another, factors cited as critical in creating peer norms and behaviors, including attitudes about sexual risk behaviors [40]. Findings highlight further opportunities to optimize support in such forums as a public health or primary care strategy. However, it has been noted that interventions must be well connected to the networks in which they are conducted [29]. Thus, it is difficult to determine whether health care professionals and health promotion practitioners would be readily allowed into this forum or others like it in expert roles to share information.

Consequently, while peer influencers and educators can be used for diffusion of messages and information to others in the forum and wider expatriate or traveler community, influence is best done indirectly. Health-promoting organizations could work to influence those who hold key positions within the forum to amplify the visibility of timely and accurate information and advice about HIV and other STIs. The use of opinion leaders working with health professionals is a strategy that has demonstrated utility in peer influence interventions used to respond to HIV, other STIs, and bloodborne viruses among men who have sex with men, sex workers, and people who inject drugs [40,43,84].

Based on our findings, an intervention using these forums can leverage positive norms around risk and relationships and increase the social capital of expatriates and travelers, including disaffected risk takers. Intervention design should provide opportunities to examine risk scenarios and provide specific information and education about the context of unsafe sexual behavior in countries with a high prevalence of HIV and other STIs, as well as promoting social connectedness [8].

#### Context of Intervention Design

Peer influence methods have been generally most successful and sustainable when driven and undertaken by peers who were part of the community and supported by broader health promotion strategies [24,42]. Peer leaders in these contexts would also engage with the broader stigma, discrimination, and rights-based issues that underpin effective prevention of HIV, other bloodborne viruses, and STIs [24,42,85].

Accordingly, in considering the amenability of such a model, health-promoting organizations and commissioning agencies must pay attention to whether the common attitudes or cultures of such online communities are compatible with an overall health promotion and rights-based approach. This is a challenge highlighted in both historical and contemporary gay community programs where significant work has been undertaken in peer programs to reduce structural and community inequities, including stigma toward people living with HIV, racism, and sexism [86]. In the current context, issues of race and gender-related stigma require further exploration in the design of interventions.

Interventions using forums should be developed in partnership with, or supportive of, local organizations; complement any in situ interventions in expatriate or long-term traveler destinations; and support information provided to expatriates and travelers via social marketing or in primary care. Support for local services may need to be considered for any increased use of health services as a result of better awareness of risks promoted via the forums. It may be that costs to destination countries prove minimal, with anecdotal evidence that expatriates and travelers seek health care in their country of origin for issues such as HIV and other STIs, but they should be factored into intervention design.

#### Evaluation and Research

Policy makers should commission further research to expand the findings of this study and better understand the way in which expatriate and traveler networks function (both online and offline), specifically the cohesion, density, and homophily of networks [87,88]. A social network analysis within and between forums would complement our findings. We recommended that research and evaluation be undertaken of a formal Web-based outreach intervention. The forum may also be considered as a space to develop, test, implement, or evaluate safer sex messages for an online component of broader campaigns or to promote testing and treatment options, including treatment as prevention.

Interventions require appropriate funding and must be of sufficient duration and dose to see positive outcomes. Policy makers should work with health-promoting organizations and researchers to develop effective indicators of impact and strategies to disseminate findings widely, preventing where possible duplication of interventions and research and allowing positive findings to be adapted or adopted for other contexts, for other health issues, or at scale. The cost effectiveness of such interventions should also be established [8].

### Conclusions

Online communities of expatriates and travelers sustain and facilitate social ties; they make geographically distant places more proximal, linking dispersed peoples to their country of origin, as well as to others in the diaspora. Whether explicitly for health or not, such forums influence and affect social connectedness, help seeking, and other health behaviors, both positively and negatively. We conclude that, to access mobile populations vulnerable to acquiring HIV and other STIs but located outside the jurisdiction of specific countries, sexual health policy makers and health-promoting organizations should use such forums to extend the reach of public health interventions. When sensitive and appropriate engagement are used, these forums provide a valuable setting to engage a priority population, provide supplementary sources of support and information, and complement other strategies to prevent or reduce the impact of HIV or other STI transmission in mobile populations.

## Abbreviations

STI: sexually transmissible infection
